# The ASM Journals Committee Values the Contributions of Black Microbiologists

**DOI:** 10.1128/microbiolspec.edt-0001-2020

**Published:** 2020-07-31

**Authors:** Patrick D. Schloss, Melissa Junior, Rebecca Alvania, Cesar A. Arias, Andreas Baumler, Arturo Casadevall, Corrella Detweiler, Harold Drake, Jack Gilbert, Michael J. Imperiale, Susan Lovett, Stanley Maloy, Alexander J. McAdam, Irene L. G. Newton, Michael Sadowsky, Rozanne M. Sandri-Goldin, Thomas J. Silhavy, Peter Tontonoz, Jo-Anne H. Young, Craig E. Cameron, Isaac Cann, A. Oveta Fuller, Ariangela J. Kozik

**Affiliations:** Chair, ASM Journals Committee; Department of Microbiology and Immunology, University of Michigan, Ann Arbor, Michigan, USA; Director, ASM Journals; American Society for Microbiology, Washington, DC, USA; Assistant Director, ASM Journals; American Society for Microbiology, Washington, DC, USA; Editor in Chief, Antimicrobial Agents and Chemotherapy; Center for Antimicrobial Resistance and Microbial Genomics and Division of Infectious Diseases, University of Texas Health Science Center, McGovern Medical School, Houston, Texas, USA; Department of Microbiology and Molecular Genetics, University of Texas Health Science Center, McGovern Medical School, Houston, Texas, USA, Houston, Texas, USA; Editor in Chief, Infection and Immunity; Department of Medical Microbiology and Immunology, University of California, Davis, California, USA; Editor in Chief, mBio; Department of Molecular Microbiology and Immunology, Johns Hopkins Bloomberg School of Public Health, Baltimore, Maryland, USA; Editor in Chief, Microbiology and Molecular Biology Reviews; Department of Molecular, Cellular & Developmental Biology, University of Colorado, Boulder, Colorado, USA; Editor in Chief, Applied and Environmental Microbiology; Department of Ecological Microbiology, University of Bayreuth, Bayreuth, Germany; Editor in Chief, mSystems; Department of Pediatrics, University of California, San Diego, California, USA; Editor in Chief, mSphere; Department of Microbiology and Immunology, University of Michigan, Ann Arbor, Michigan, USA; Editor in Chief, EcoSal Plus; Department of Biology, Brandeis University, Waltham, Massachusetts, USA; Editor in Chief, Journal of Microbiology and Biology Education (JMBE); Department of Biology, San Diego State University, San Diego, California, USA; Editor in Chief, Journal of Clinical Microbiology; Boston Children’s Hospital, Boston, Massachusetts, USA; Harvard Medical School, Boston, Massachusetts, USA; Editor in Chief, Microbiology Resource Announcements; Department of Biology, Indiana University, Bloomington, Indiana, USA; Editor in Chief, Microbiology Spectrum; BioTechnology Institute, University of Minnesota, St. Paul, Minnesota, USA; Editor in Chief, Journal of Virology; Department of Microbiology and Molecular Genetics, University of California, Irvine, California, USA; Editor in Chief, Journal of Bacteriology; Department of Molecular Biology, Princeton University, Princeton, New Jersey, USA; Editor in Chief, Molecular and Cellular Biology; Department of Pathology and Laboratory Medicine, David Geffen School of Medicine, University of California, Los Angeles, California, USA; Editor in Chief, Clinical Microbiology Reviews; Department of Medicine, University of Minnesota, Minneapolis, Minnesota, USA; Department of Microbiology & Immunology, University of North Carolina, Chapel Hill, North Carolina, USA; Carl R. Woese Institute for Genomic Biology, University of Illinois, Urbana, Illinois, USA; Department of Microbiology and Immunology, University of Michigan, Ann Arbor, Michigan, USA; Department of Internal Medicine, University of Michigan, Ann Arbor, Michigan, USA

Black lives matter. The ongoing problem of police brutality and the resulting deaths of George Floyd (1), Breonna Taylor (2), and many other Black people in the United States (3) has rightly shaken the country. Acts of racism should cause us to question the level to which we have personally participated in the systems of racial inequity that facilitate such acts. We all have an obligation to identify the ways that systemic racism functions in our society and in science. As scientists, we prefer to believe that we are driven by data and are immune to such detrimental behaviors. Yet, if we are honest, we know that this is not always true.

Between 1932 and 1972, the horrific Tuskegee syphilis study was performed to observe the natural history of latent syphilis infection in 399 Black men (4). The premise of the study was driven by the racist pseudoscience of Social Darwinism. The study directors hypothesized that Black men were inferior to white men. The study directors lied to the men about their condition, leading the men to infect their partners and children. Furthermore, when penicillin was shown in 1947 to treat syphilis, the doctors hid the treatment and refused to treat the men. Leading peer-reviewed journals of the time published results from the study. This textbook example of racism in microbiology underscores the historic role of scientific publishers in disseminating racist ideologies and points to the potential for scientific publishers to prevent the spread of racism.

As the Journals Committee of the American Society for Microbiology, we are committed to promoting the work of Black microbiologists and the issues that impact the Black community. To do this, we must improve the representation of Black microbiologists across the peer review process, recruit Black authors to publish their research in ASM’s journals, and identify aspects of peer review where there is opportunity for bias to affect our decisions to publish their research, something that we wish to avoid. We must also reassess the scopes of our journals to ensure that the microbiological problems that are important to the Black community are published within the journals of this Society.

Issues that affect the Black community matter. Black people in the United States in 2020 are, and historically have been, disproportionately and negatively impacted by infectious diseases (5). The Flint, MI, water crisis brought significant suffering to the primarily Black community, including outbreaks of Legionnaires’ disease (6). Black women are more likely to have a preterm birth, of which half are associated with a microbial etiology (7). Black children are more likely to have asthma, a disease which is associated with increased bacterial burden in the lungs (8). Black people are more likely to have a severe case of and die from coronavirus disease 2019 (COVID-19) (9). In New York City and elsewhere, the death rate due to COVID-19 for Black people is twice that for white people (10). Black people are also far more likely to be affected by sexually transmitted infections, including HIV, and evidence suggests a role for underlying structural inequities, such as mass incarceration and unequal treatment when seeking medical care (11, 12). These disparities in health are an outcome of differences in socio-economic factors and the corresponding disenfranchisement. These include less access to health care, food deserts where nutritious and affordable food is not available, and poorly funded public health infrastructure. A person’s race provides no biological basis for the observed health disparities, and to assert otherwise will slow the identification of solutions to these disparities. Unfortunately, research related to solving such problems is often discounted. A recent analysis of research project (R01) proposals reviewed by the National Institutes of Health found that the community- and population-level research topics of interest to Black scientists placed them at a disadvantage for a fundable outcome and accounts for much of the reduced success rate of Black scientists (13). As an academic publisher, we have a responsibility to help to promote the importance and legitimacy of work that is important to the Black community.

Black scientists have made significant contributions in spite of the systemic racism that they have faced throughout their lives. These scientists should be able to put energy into their science rather than into overcoming the bias and prejudice that deters their efforts and devalues their humanity. As an example, George Washington Carver was born into slavery yet went on to become a preeminent plant biologist, chemist, and microbiologist despite many barriers to safely obtaining an education (https://www.tuskegee.edu/support-tu/george-washington-carver). He improved the lives of farmers by developing alternative crops to cotton, harnessing the power of rhizobia to help improve soil health, and fighting fungal plant pathogens. He impacted the lives of many Black and non-Black people. Numerous Black microbiologists have had significant impacts on topics that are particularly relevant to Black communities and beyond, including Drs. William Hinton, Ruth Moore, Jane Hinton, and many others. In an interview at the 2017 Microbe meeting in New Orleans, LA, Dr. Marian Johnson-Thompson (University of the District of Columbia) recounted the lives of many of these and other Black microbiologists (14). She told the story of Dr. Moore, a professor at Howard University who attended a General Meeting of the ASM. Because of segregation, she was not able to stay at hotels within the city or eat at any of its restaurants. Although we want to believe that such systemic racism no longer exists within our discipline, we must constantly question that assumption. The stories of these microbiologists emphasize that representation matters. They underscore the fact that unless the perspective and challenges of Black communities are represented, then they will not be addressed.

In a recent mSphere of Influence article, Dr. Michael Johnson recounted his shock that although there was a 930% increase in the number of biomedical Ph.D.’s awarded to Black people and those from other underrepresented groups (URGs) between 1980 and 2013, there has not been a meaningful change in the number of assistant professors from URGs over that time (15). In his article, he asks how he wound up at a research-intensive university as a Black professor. He asks two questions of himself: “by what miracle did I beat the odds to get here?” and “what can I do to get other URMs [underrepresented minorities] in a similar position as myself?” Yet, it is not Dr. Johnson’s responsibility alone to remove these barriers. As a publisher of microbiology research, we acknowledge the important role that we have in the career development of junior scientists and the role that we have in giving legitimacy to scientific questions. For too long, we have not promoted the work of junior Black and other scientists from URGs as much as we could have. We have been too passive in recruiting these scientists to publish in our journals. Scientists like Dr. Johnson should not think that their success is a “miracle.” We also must ask ourselves what we can do to get more scientists from URGs into faculty and leadership positions. Although we should always strive to recruit more people from URGs into science, the data that he reports indicate that the problem also lies with retention of this talent. As leaders of the ASM Journals program, we need to take a greater role in mentorship. We can recruit more junior scientists from URGs to be peer reviewers, put them in leadership positions, and publicly recognize them.

As the Journals Committee, we seek to improve the representation of Black microbiologists and therefore take on the responsibility to do the following.

- Learn from the stories of Black microbiologists, past and present. We will listen. Black microbiologists should not have to shoulder the burden of dismantling systems of inequality on their own.
- Ensure that diverse voices and viewpoints are represented among the editors in chief. We will conduct open searches that actively recruit Black scientists and scientists from URGs. We will not constrain the candidate pool to current or past editors and Editorial Board members, which have traditionally been the source of candidates.
- Appoint editors in chief who understand that the impact of their journal is dependent on the diversity of their authors, reviewers, and editors. We will ask candidates to state their experience fostering diversity, equity, and inclusion in their application.
- Improve the representation of Black scientists and those from other URGs across the peer review system. Each journal will develop a plan that will be regularly evaluated and used as a criterion to determine whether an editor in chief should be reappointed.
- Be alert to implicit and overt bias when handling manuscripts from Black and other scientists from URGs. We will work with our editors and others to understand bias and study where it can manifest itself in peer review.
- More fully represent the scope of how microbiology impacts the Black community. We will solicit input from Black microbiologists for topics that are not being addressed adequately within ASM’s journals and revise the scopes of the journals accordingly.
- Promote Black microbiologists. We will ensure that their representation is equitable when selecting papers for editor spotlights, authors for commentaries, and subjects for biographical reports.
- Help develop the next generation of microbiologists and more actively listen. We will participate in opportunities to serve and mentor Black scientists and those from other URGs through the Annual Biomedical Research Conference for Minority Students (ABRCMS) and the annual conference for the Society for Advancement of Chicanos/Hispanics & Native Americans in Science (SACNAS).
- Identify appropriate methods for identifying and quantifying representation of Black microbiologists. We will collaborate closely with the ASM Taskforce on Diversity, Equity, and Inclusion.

There is no place for anti-Black or for any form of racism within microbiology. To solve the most important microbiological problems of today and prepare for those of the future, we must leverage the experiences, perspectives, and expertise of everyone.
